# Protein Distribution during Human Erythroblast Enucleation *In Vitro*

**DOI:** 10.1371/journal.pone.0060300

**Published:** 2013-04-02

**Authors:** Amanda J. Bell, Timothy J. Satchwell, Kate J. Heesom, Bethan R. Hawley, Sabine Kupzig, Matthew Hazell, Rosey Mushens, Andrew Herman, Ashley M. Toye

**Affiliations:** 1 School of Biochemistry, University of Bristol, Bristol, United Kingdom; 2 Bristol Institute of Transfusion Science, NHS Blood and Transplant, Bristol, United Kingdom; Southern Illinois University School of Medicine, United States of America

## Abstract

Enucleation is the step in erythroid terminal differentiation when the nucleus is expelled from developing erythroblasts creating reticulocytes and free nuclei surrounded by plasma membrane. We have studied protein sorting during human erythroblast enucleation using fluorescence activated cell sorting (FACS) to obtain pure populations of reticulocytes and nuclei produced by *in vitro* culture. Nano LC mass spectrometry was first used to determine the protein distribution profile obtained from the purified reticulocyte and extruded nuclei populations. In general cytoskeletal proteins and erythroid membrane proteins were preferentially restricted to the reticulocyte alongside key endocytic machinery and cytosolic proteins. The bulk of nuclear and ER proteins were lost with the nucleus. In contrast to the localization reported in mice, several key erythroid membrane proteins were detected in the membrane surrounding extruded nuclei, including band 3 and GPC. This distribution of key erythroid membrane and cytoskeletal proteins was confirmed using western blotting. Protein partitioning during enucleation was investigated by confocal microscopy with partitioning of cytoskeletal and membrane proteins to the reticulocyte observed to occur at a late stage of this process when the nucleus is under greatest constriction and almost completely extruded. Importantly, band 3 and CD44 were shown not to restrict specifically to the reticulocyte plasma membrane. This highlights enucleation as a stage at which excess erythroid membrane proteins are discarded in human erythroblast differentiation. Given the striking restriction of cytoskeleton proteins and the fact that membrane proteins located in macromolecular membrane complexes (e.g. GPA, Rh and RhAG) are segregated to the reticulocyte, we propose that the membrane proteins lost with the nucleus represent an excess mobile population of either individual proteins or protein complexes.

## Introduction

During the final stages of erythroid terminal differentiation, the orthochromatic erythroblast enucleates to form the reticulocyte. Whilst undergoing this dramatic process, erythroid membrane proteins, cytoskeletal proteins and other cellular machinery required by the nascent reticulocyte must be selectively retained or will be lost with the extruded nucleus [1]. Studies using mouse erythroblasts have shown that the spectrin cytoskeleton, along with microtubules, myosin and actin partitions to the reticulocyte as the nucleus is removed [2], [3], [4]. Key erythroid membrane surface proteins were observed to be segregated to the nascent reticulocyte following enucleation including band 3 [5], [6], GPA [5], GPC [5] and RhAG [5] in murine cells. Several membrane proteins are selectively lost, such as the Beta 1 integrin [7], the vitamin C transporter SVCT2 [8] and erythroblast macrophage protein (EMP) [7], [9]. A mechanism has been proposed whereby retention of erythrocyte membrane proteins occurs by attachment to the cytoskeletal network via associated adaptor proteins or indirectly via multiprotein membrane protein complexes comprising band 3 or GPC [10]. Supporting this hypothesis, GPA cytoskeletal attachment is greater in erythroblasts than in reticulocytes [7], and the disruption of cytoskeletal attachment in ankyrin and protein 4.1R knockout mice resulted in the mislocalisation of specific membrane proteins (band 3 and RhAG for ankyrin disruption and GPC for protein 4.1) to the plasma membrane surrounding the nucleus [5].

It is currently unknown whether the protein sorting mechanism during enucleation is similar in humans. Griffiths et al recently presented confocal images of a selected number of membrane proteins, including GPA, GPC and Rh. Some immunofluorescence surrounding the extruding nucleus was perceivable in the images presented, and both basigin and beta 1 integrin were lost along with the nucleus [11]. However, partitioning of the majority of key erythrocyte membrane proteins (e.g. band 3, RhAG, Glut1, CD44) and many cytoskeletal proteins (alpha and beta spectrin, ankyrin or protein 4.2) was not investigated. We hypothesized that since differences in membrane protein multiprotein complex composition are known to exist between humans and mice [10], subtle differences may exist in the sorting process that occurs during enucleation. Identifying potential disparities is important to fully understand how specific protein deficiencies occur in human red blood cell diseases such as Hereditary Spherocytosis.

This study has adopted a global proteomic approach in combination with biochemical and detailed immunofluorescence analysis to explore the protein distribution and partitioning that occurs during human erythroblast enucleation. In general we find that there is a preferential restriction of erythroid membrane proteins to the reticulocyte and that this partitioning occurs at a very late stage during enucleation. Importantly, a substantial proportion of some membrane proteins, in particular band 3, CD44, GPC, Glut1 and stomatin are lost in the plasma membrane surrounding the nucleus in humans.

## Methods

### Antibodies

Monoclonal mouse antibodies used were BRIC256 (GPA), BRIC170 (band 3), LA1818 (RhAG), BRIC69 (Rh), BRIC4 (GPC), BRIC272/BRIC274 (ankyrin), BRIC273 (protein 4.2), BRAC65 (beta spectrin), BRIC172/BRIC276 (alpha spectrin), BRIC32 (CD47) (IBGRL, Filton, Bristol, UK), beta actin (Sigma), PDI (Assay Designs) and calnexin (RDI). BRIC272, BRIC273, BRIC276 and BRAC65 are all novel unpublished monoclonal antibodies. The novel antibodies were characterised using GFP-tagged cDNA expression, shRNA knockdown in K562 cells, and by using mature erythrocytes with a known protein deficiency. Rabbit monoclonal antibody used was beta 1 integrin (Novus). Rabbit polyclonal antibodies used were band 3, RhAG, GPC, Rh, Glut1, protein 4.1, p55, stomatin and CD44 (all available in house), flotillin-2 (Cell Signalling), alpha adducin (Santa Cruz). A goat polyclonal to lamin B was purchased from Santa Cruz. Secondary antibodies used were goat anti–mouse-Alexa 488 and goat anti-rabbit-Alexa 594 (Invitrogen), rabbit anti-mouse RPE, HRP-conjugated swine anti-rabbit and rabbit anti-mouse (Dako) and HRP conjugated donkey anti-goat (Jackson ImmunoResearch).

### Erythroblast Cell Culture

Peripheral blood mononuclear cells were isolated from platelet apheresis waste blood (NHSBT, Bristol) from healthy donors with written informed consent for research use in accordance with the Declaration of Helsinki and approved by local Research Ethics Committee (Southmead Research Ethics Committee reference 08/H0102/26 and Bristol Research Ethics Committee Centre reference 12/SW/0199). Erythroblasts were expanded and differentiated using either the whole population of Peripheral Blood Mononuclear cells or from CD34+ as described previously [11], [12], [13]. The culture method for the PBMC population was modified as follows; a lineage depletion step (Lineage Cell Depletion Kit, Miltenyi Biotec, UK) was performed following Percoll on day 5 to ensure complete removal of lineage positive cells at this stage. IMDM (Source Biosciences) supplemented with 2% (v/v) fetal bovine serum (Hyclone, Fisher Scientific UK Ltd), 10 µg/ml insulin (Sigma), 200 µg/ml holotransferrin (Sigma), 3% (v/v) AB serum (Sigma) and 3 U/ml heparin (Sigma) replaced StemSpan SFEM during the expansion (phase 2) and differentiation phases (phase 3). Therefore, during Phase 2 of the culture IMDM base medium was supplemented with 2 U/ml Epo (Bristol Royal Infirmary, Bristol, UK), 1 µM dexamethasone (Sigma), 40 ng/ml IGF-1 (R&D systems), 40 µg/ml cholesterol-rich lipids (Sigma) and SCF (100 ng/ml). For Phase 3 of the culture, IMDM base medium was supplemented with 10 U/ml Epo, 1 mg/ml holotransferrin (Sigma), 3% human AB plasma (Sigma), 10 µg/ml insulin (Sigma), 1 µM thyroid hormone (Sigma), 40 ng/ml IGF-1, and 40 µg/ml cholesterol-rich lipids.

### FACS Sorting

5×10^7^ batches of enucleating erythroblasts were washed with PBS, then dual labelled with Hoechst 33342 (5 µg/ml) (Sigma) and BRIC256 (GPA) (detected with PE conjugated secondary). The reticulocyte and nuclei populations were then sorted using a BD Influx Cell Sorter. 1×10^5^ cells from each population were cytospun as previously described [13]. The reticulocyte or nuclei populations were pelleted and stored at −80°C.

### Proteomics

1×10^6^ reticulocytes or nuclei were fractionated by 1D SDS-PAGE, gel lanes were cut into 4 equal portions and in-gel digested with trypsin. Extracted peptides were subjected to Nano LC mass spectrometry as described [14] but with modifications. The raw data files were processed using Proteome Discoverer software v1.2 (Thermo Scientific) and searched against the UniProt/SwissProt Human database release version 57.3 (20326 entries) using the SEQUEST (Ver. 28 Rev. 13) algorithm. Peptide precursor mass tolerance was set at 10 ppm, and MS/MS tolerance was set at 0.8 Da. Search criteria included carbamidomethylation of cysteine (+57.0214) as a fixed modification and oxidation of methionine (+15.9949) as a variable modification. Searches were performed with full tryptic digestion and a maximum of 1 missed cleavage was allowed. The reverse database search option was enabled and all peptide data was filtered to satisfy false discovery rate (FDR) of 5%. The Proteome Discoverer software generates a reverse “decoy” database from the same protein database and any peptides passing the initial filtering parameters that were derived from this decoy database are defined as false positive identifications. The minimum cross-correlation factor (Xcorr) filter was readjusted for each individual charge state separately to optimally meet the predetermined target FDR of 5% based on the number of random false positive matches from the reverse decoy database. Thus each data set has its own passing parameters.

### Immunofluorescence

Immunostaining of enucleating erythroblasts were conducted as described previously [13]. Briefly, 6 × 10^5^ cells were fixed in suspension in 0.5% acrolein in PBS (Sigma-Aldrich), washed 3 times in PBS-0.1M glycine before being cytospun onto coverslips coated with Cell-Tak (BD Biosciences). Cells were then permeabilized with 0.05% Triton X-100 for 5 minutes at room temperature and then blocked in PBS-4% BSA for 45 minutes, incubated with primary antibodies in PBS-4% BSA for 1 hour, washed with PBS, and incubated for 1 hour with goat anti–mouse Alexa 488–conjugated (Invitrogen) secondary antibodies and 4′,6-diamidino-2-phenylindole (Invitrogen). Coverslips were washed and mounted on microscope slides using Mowiol (Calbiochem) containing 2.5% (w/v) Dabco antifade reagent (Sigma-Aldrich). Confocal images were taken using a Leica AOBS SP2 confocal microscope (63×/1.4 NA oil-immersion lens and processed using Adobe Photoshop 9.0).

### SDS-PAGE and Western Blotting

0.5−1×10^6^ cells were lysed for 10 min on ice in lysis buffer (20 mM Tris-HCl, pH 8.0, 137 mM NaCl, 10 mM EDTA, 100 mM NaF, 1% (v/v) Nonidet P-40, 10% (v/v) glycerol, 10 mM Na_3_VO_4_, 2 mM PMSF and protease inhibitors, Calbiochem). Omnicleave (10 U/µl, Epicentre) was added to lysis buffer supplemented with 10 mM MgCl_2_ to digest the DNA present in the nuclei pellets. Equal numbers of lysed reticulocytes and nuclei were loaded and separated by SDS-PAGE and then immunoblotted.

## Results

### Protein Distribution in Reticulocyte and Nuclei Populations by Proteomics

**Figure 1:**
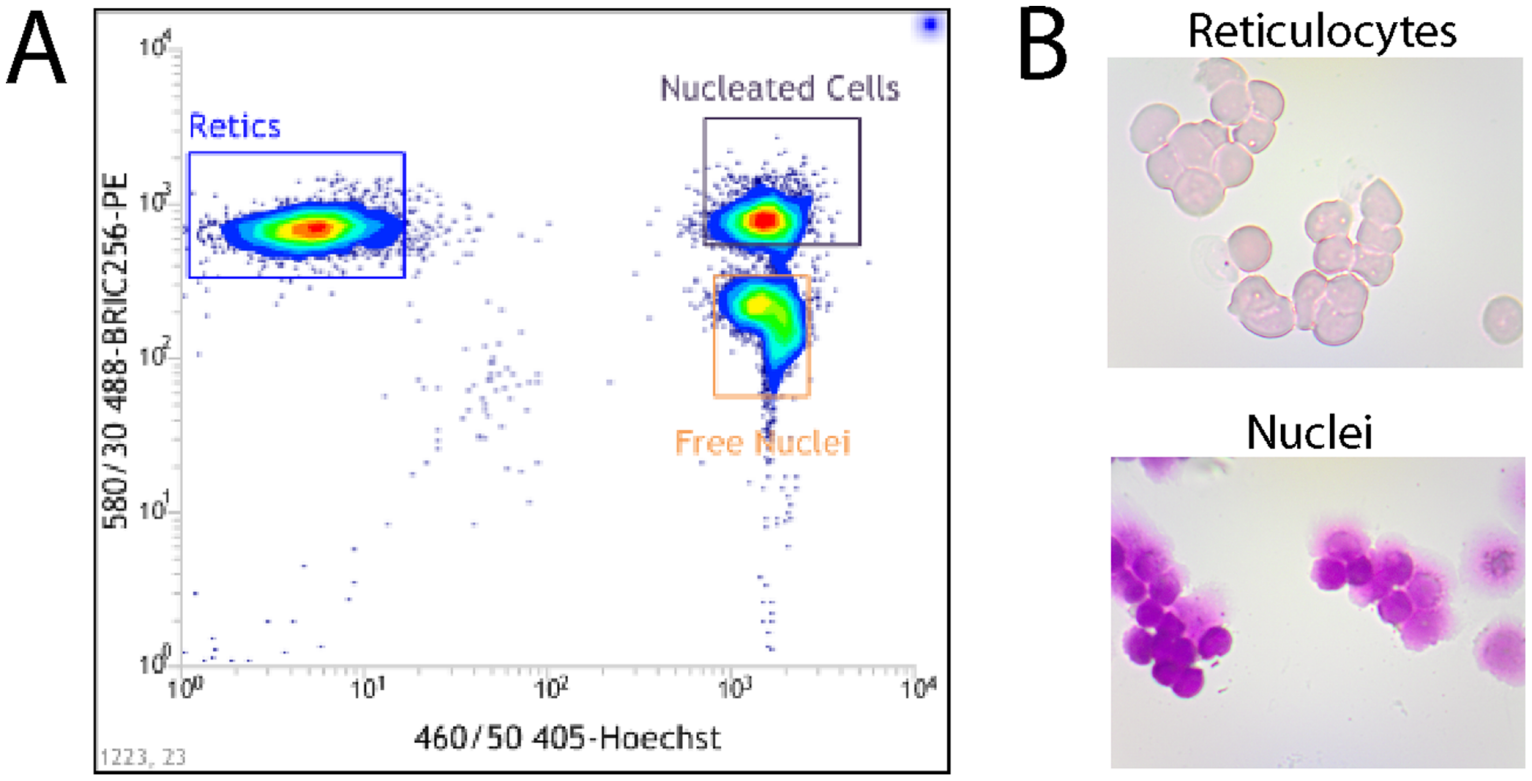
FACs sorting of reticulocytes and extruded nuclei. A) Extruded nuclei and reticulocytes were separated by fluorescence activated cell sorting based on fluorescence intensity of DNA (Hoechst) and GPA (BRIC256) staining as outlined in the Materials and Methods. B) Representative cytospins from the sorted reticulocyte (upper panel) and extruded nuclei (lower panel) populations are shown.

GPA expression combined with Hoechst staining was exploited to separate reticulocytes and extruded nuclei [15] produced by *in vitro* erythroblast culture [11], [12], [13]. Three discrete populations were identified by flow cytometry; the reticulocyte population (GPA^high^:Hoechst^negative^), extruded nuclei (GPA^low^:Hoechst^positive^) and nucleated erythroblasts (GPA^high^, Hoechst^positive^) (Figure 1A). These populations were isolated using FACS sorting, and cytospins (Figure 1B) confirmed the purity of the reticulocyte (95.3+/−0.65% (n = 3, +/− SEM)) and nuclei (96.9+/−0.56% (n = 3,+/− SEM)) populations.

To determine the protein distribution during enucleation, a proteomic comparison of the reticulocyte and extruded nuclei populations was undertaken. Tables 1–4 show a summarised list of peptides detected in the reticulocyte and nuclei populations. Table 1 shows membrane protein peptides detected, Table 2 shows examples of cytoskeletal or cytoskeletal interacting proteins detected, Table 3 nuclear and ER proteins and Table 4 cytosolic proteins and endocytic machinery. As expected, reticulocytes were enriched for peptides of cytoskeletal and erythrocyte membrane proteins. In addition a host of peptides derived from proteins from other cellular compartments such as cytosolic enzymes and endocytic proteins (e.g. Lamp1, clathrin, adaptor proteins, dynamin, sorting nexins,) were enriched in reticulocytes.

**Table 1:** Proteomic profile of membrane protein distribution in sorted populations of reticulocytes and extruded nuclei.

		Nuclei		Reticulocytes	
Accession	Description	Total peptides	Unique peptides	Total peptides	Unique peptides
Q9HDC9	Adipocyte plasma membrane-associated protein	22	12	6	4
Q02094	Ammonium transporter Rh type A	3	2	6	2
B4DNW4	Aquaporin 1	9	3	12	4
Q5T5M0	Aquaporin 7	4	1	3	1
Q9NP58	ATP-binding cassette sub-family B member 6, mitochondrial	4	4	40	18
P02730	Band 3 anion transport protein	195	28	477	36
Q54A51	Basigin	36	8	32	8
B6EAT9	CD44	2	2	4	2
E9PB22	CD47			3	1
Q99808	Equilibrative nucleoside transporter 1	11	5	14	5
Q96PL5	Erythroid membrane-associated protein			14	7
O75955	Flotillin-1	9	8	46	20
Q14254	Flotillin-2	6	5	42	20
P11166	Glucose transporter, type 1	51	10	78	12
P04921	Glycophorin-C	13	2	29	3
Q86SU0	Immunoglobulin-like domain-containing receptor 1	9	1	1	1
P20702	Integrin alpha-X	6	2	1	1
P05556	Integrin beta-1	11	7	2	2
P23276	Kell blood group glycoprotein			6	4
O75387	Large neutral amino acids transporter small subunit 3	2	1	4	2
P51811	Membrane transport protein XK			4	3
O15173	Membrane-associated progesterone receptor component 2	25	6	4	2
P53985	Monocarboxylate transporter 1	7	4	6	4
O15439	Multidrug resistance-associated protein 4	2	2	12	10
Q6PIU2	Neutral cholesterol ester hydrolase 1	52	16	12	7
P20020	Plasma membrane calcium-transporting ATPase 1	7	5	14	11
Q16720	Plasma membrane calcium-transporting ATPase 3	6	4	9	7
P23634	Plasma membrane calcium-transporting ATPase 4	12	10	20	16
Q9Y4D8	Probable E3 ubiquitin-protein ligase C12orf51	1	1	44	33
Q5VSJ9	Rh blood group, CcEe antigens	3	2	8	4
E9PS74	SLC43A3	3	2	4	3
Q96QG1	Sodium/calcium exchanger SCL8A3			2	2
B7Z3U6	Sodium/potassium-transporting ATPase subunit alpha-1	14	9	14	12
P54709	Sodium/potassium-transporting ATPase subunit beta-3	4	3	2	2
P27105	Stomatin	118	16	126	16
Q9UJZ1	Stomatin-like protein 2	5	4	13	11
Q9H1E5	Thioredoxin-related transmembrane protein 4	3	2		
A6NJC0	TMCC2	65	13	18	6
P02786	Transferrin receptor protein 1	81	26	75	25
B7Z1P7	Transmembrane and coiled-coil domain family 2	151	25	36	15
Q13336	Urea transporter 1			2	2
Q9Y6M5	Zinc transporter 1	2	1	4	4

Sorted populations of reticulocytes and extruded nuclei were fractionated by 1D SDS-PAGE and subjected to Nano LC mass spectrometry. An abridged list containing key erythroid membrane proteins of interest is shown. Total peptide column is the total number of peptides (and therefore an indication of a particular protein’s abundance) detected in the population, whilst the unique peptide column indicates the number of unique peptides detected. To assess differences between nuclei and reticulocyte populations the total peptide number should be used.

**Table 2:** Proteomic profile of erythroid cytoskeletal protein distribution in sorted populations of reticulocytes and extruded nuclei.

		Nuclei		Reticulocytes	
Accession	Description	Total peptides	Unique peptides	Total peptides	Unique peptides
Q00013	55 kDa erythrocyte membrane protein	27	15	93	27
P68032	Actin, alpha cardiac muscle	116	16	127	15
P60709	Actin, cytoplasmic	223	25	228	24
P61160	Actin-related protein 2	6	5	22	11
O15143	Actin-related protein 2/3 complex subunit 1B	2	2	14	7
O15144	Actin-related protein 2/3 complex subunit 2	4	3	25	11
P61158	Actin-related protein 3	12	9	44	15
O43707	Alpha-actinin-4	4	4	12	7
P35611	Alpha-adducin	9	5	78	20
P16157	Ankyrin-1	123	65	476	104
E9PE32	Ankyrin-3	8	5	31	9
Q562R1	Beta-actin-like protein 2	41	6	46	6
P35612	Beta-adducin			72	25
B1AK87	Capping protein (Actin filament) muscle Z-line, beta	10	4	43	12
Q96H99	Cortactin	1	1	20	13
Q08495	Dematin	16	10	83	21
A8K8J9	Dynactin 2 (P50), isoform CRA_b	6	5	32	16
Q4KKX0	Erythrocyte membrane protein band 4.2	30	13	164	40
P21333	Filamin-A	27	23	94	66
Q9UEY8	Gamma-adducin			14	7
A2A418	Gelsolin	23	9	15	7
P33176	Kinesin-1 heavy chain			17	12
Q15691	Microtubule-associated protein RP/EB family member 1	5	3	12	8
P12829	Myosin light chain 4	10	5	24	8
P60660	Myosin light polypeptide 6	3	1	4	3
Q3MIV8	Myosin, heavy chain 11, smooth muscle	12	9	43	19
P35580	Myosin-10	48	38	210	106
Q7Z406	Myosin-14	10	8	25	12
P35579	Myosin-9	75	54	373	124
P11171	Protein 4.1	38	17	159	33
P02549	Spectrin alpha chain, erythrocyte	138	87	735	176
P11277	Spectrin beta chain, erythrocyte	105	70	637	165
Q9Y490	Talin-1	66	48	224	102
Q9Y4G6	Talin-2	6	6	24	11
P28289	Tropomodulin-1	4	4	32	16
D9YZV5	Tropomyosin 1 (Alpha) isoform 4	9	5	10	4
Q5VU58	Tropomyosin 3	19	9	37	12
P06753	Tropomyosin alpha-3 chain	11	6	18	6
Q71U36	Tubulin alpha-1 chain	61	13	141	21
P07437	Tubulin beta chain	89	23	249	28
A8MUB1	Tubulin, alpha 1 (Testis specific)	49	9	119	20
B3KPW9	Tubulin, alpha 8	33	7	80	13
B3KS31	Tubulin, beta 6	23	7	61	9
P18206	Vinculin	9	6	57	35

Sorted populations of reticulocytes and extruded nuclei were fractionated by 1D SDS-PAGE and subjected to Nano LC mass spectrometry. An abridged list containing key cytoskeletal proteins of interest is shown. Total peptide column is the total number of peptides (and therefore an indication of a particular protein’s abundance) detected in the population, whilst the unique peptide column indicates the number of unique peptides detected. To assess differences between nuclei and reticulocyte populations the total peptide number should be used.

**Table 3:** Proteomic profile of nuclear and ER protein distribution in sorted populations of reticulocytes and extruded nuclei.

		Nuclei		Reticulocytes	
Accession	Description	Total peptides	Unique peptides	Total peptides	Unique peptides
P11021	78 kDa glucose-regulated protein	96	30	48	24
P46013	Antigen KI-67	262	132	14	12
O00148	ATP-dependent RNA helicase DDX39A	57	19	18	9
Q8IWX8	Calcium homeostasis endoplasmic reticulum protein	3	3		
P27824	Calnexin	45	15	8	5
P27797	Calreticulin	95	16	27	13
P11387	DNA topoisomerase 1	129	33	2	2
P78527	DNA-dependent protein kinase catalytic subunit	194	113	55	47
O60762	Dolichol-phosphate mannosyltransferase	21	15		
P39656	Dolichyl-diphosphooligosaccharide–protein glycosyltransferase 48 kDa subunit	20	11	4	4
P04843	Dolichyl-diphosphooligosaccharide–protein glycosyltransferase subunit 1	56	26	9	7
P49792	E3 SUMO-protein ligase RanBP2	61	47		
Q9NZ08	Endoplasmic reticulum aminopeptidase 1	4	4		
P30040	Endoplasmic reticulum resident protein 29	20	8	3	1
Q9BS26	Endoplasmic reticulum resident protein 44	16	9	6	3
Q969X5	Endoplasmic reticulum-Golgi intermediate compartment protein 1	3	3		
P14625	Endoplasmin	33	22	12	9
Q9P0I2	ER membrane protein complex subunit 3	3	3		
O75396	ER-Golgi SNARE of 24 kDa	40	11	8	5
Q9Y5B9	FACT complex subunit SPT16	62	30	4	4
A8K318	Glucosidase 2 subunit beta	23	13	6	6
P09601	Heme oxygenase 1	16	9		
Q9BXL5	Hemogen	119	21	42	16
Q5SSJ5	Heterochromatin protein 1-binding protein 3	40	18	1	1
P09429	High mobility group protein B1	160	16	14	5
P26583	High mobility group protein B2	188	17	23	10
Q02539	Histone H1.1	144	10	18	4
P04908	Histone H2A type 1-B/E	182	6	14	4
P68431	Histone H3.1	117	12	22	5
P62805	Histone H4	266	14	33	10
Q5TCI8	Lamin A/C	212	43	40	19
P42166	Lamina-associated polypeptide 2, isoform alpha	148	34	11	5
P42167	Lamina-associated polypeptide 2, isoforms beta/gamma	137	22	13	7
Q14739	Lamin-B receptor	53	17	2	2
P20700	Lamin-B1	162	43	22	13
Q03252	Lamin-B2	103	35	5	4
P43243	Matrin-3	28	13	5	2
Q8N4V1	Membrane magnesium transporter 1	2	1		
Q9UNW1	Multiple inositol polyphosphate phosphatase 1	25	14	5	5
Q8NFW8	N-acylneuraminate cytidylyltransferase	111	24	8	4
Q14697	Neutral alpha-glucosidase AB	88	32	22	14
Q8N1F7	Nuclear pore complex protein Nup93	33	18	6	4
Q8TEM1	Nuclear pore membrane glycoprotein 210	60	33	2	2
Q9NR30	Nucleolar RNA helicase 2	39	20		
P19338	Nucleolin	99	32	18	15
Q5SRE5	Nucleoporin NUP188 homolog	15	13	1	1
P12270	Nucleoprotein TPR	105	68	17	12
P02545	Prelamin-A/C	284	56	54	26
P07237	Protein disulfide-isomerase	74	22	27	16
P13667	Protein disulfide-isomerase A4	7	7		
B7Z254	Protein disulfide-isomerase A6	24	12	7	5
P49257	Protein ERGIC-53	3	3	3	2
Q5JYR6	Ribophorin II	31	10	5	3
P55072	Transitional endoplasmic reticulum ATPase	57	25	133	50
Q9NYU2	UDP-glucose:glycoprotein glucosyltransferase 1	75	45	28	19
O95292	Vesicle-associated membrane protein-associated protein B/C	11	3	2	1

Sorted populations of reticulocytes and extruded nuclei were fractionated by 1D SDS-PAGE and subjected to Nano LC mass spectrometry. An abridged list containing key nuclear proteins and ER proteins of interest is shown. Total peptide column is the total number of peptides (and therefore an indication of a particular protein’s abundance) detected in the population, whilst the unique peptide column indicates the number of unique peptides detected. To assess differences between nuclei and reticulocyte populations the total peptide number should be used.

**Table 4:** Proteomic profile of cytosolic and endocytic protein distribution in sorted populations of reticulocytes and extruded nuclei.

		Nuclei		Reticulocytes	
Accession	Description	Total peptides	Unique peptides	Total peptides	Unique peptides
P62258	14-3-3 protein epsilon	43	16	85	20
P61981	14-3-3 protein gamma	26	12	34	11
P63104	14-3-3 protein zeta/delta	35	12	50	11
Q01813	6-phosphofructokinase type C	1	1	47	21
B4DQJ8	6-phosphogluconate dehydrogenase, decarboxylating	24	12	84	24
P49588	Alanyl-tRNA synthetase, cytoplasmic	13	10	93	37
Q10567	AP-1 complex subunit beta-1	3	3	28	21
O95782	AP-2 complex subunit alpha-1	6	6	69	32
P63010	AP-2 complex subunit beta	6	5	46	27
Q2M2I8	AP2-associated protein kinase 1			5	5
C9JPM4	ARF4	23	6	7	4
P53396	ATP-citrate synthase	26	16	166	49
P07738	Bisphosphoglycerate mutase	34	13	78	17
P11586	C-1-tetrahydrofolate synthase, cytoplasmic	26	17	141	48
P07384	Calpain-1 catalytic subunit	7	6	78	36
P00915	Carbonic anhydrase 1	32	11	64	14
P00918	Carbonic anhydrase 2	49	15	92	21
P04040	Catalase	95	32	376	47
Q00610	Clathrin heavy chain 1	49	35	243	80
P53675	Clathrin heavy chain 2	6	6	40	17
P30046	D-dopachrome decarboxylase	1	1	7	5
Q16531	DNA damage-binding protein 1	41	22	89	48
P46734	Dual specificity mitogen-activated protein kinase kinase 3	33	15	57	20
P50570	Dynamin-2	3	3	23	16
E9PD66	E3 ubiquitin-protein ligase HUWE1	12	7	118	84
Q15075	Early endosome antigen 1	2	2	1	1
P13639	Elongation factor 2	23	11	84	32
P60842	Eukaryotic initiation factor 4A-I	52	18	90	23
P49327	Fatty acid synthase	19	15	135	76
P30043	Flavin reductase	123	16	253	19
P04075	Fructose-bisphosphate aldolase A	55	20	181	31
P11413	Glucose-6-phosphate 1-dehydrogenase	13	9	84	31
P48506	Glutamate–cysteine ligase catalytic subunit	4	4	81	31
E7EU54	Glyceraldehyde-3-phosphate dehydrogenase	42	11	104	14
P49840	Glycogen synthase kinase-3 alpha	3	3	7	4
P08107	Heat shock 70 kDa protein 1A/1B	77	23	96	32
P34932	Heat shock 70 kDa protein 4	10	7	77	35
P17066	Heat shock 70 kDa protein 6	39	9	35	8
P07900	Heat shock protein HSP 90-alpha	114	35	177	45
P08238	Heat shock protein HSP 90-beta	77	31	94	33
P54652	Heat shock-related 70 kDa protein 2	61	11	54	12
P69905	Hemoglobin subunit alpha	532	14	713	17
P68871	Hemoglobin subunit beta	802	20	1199	21
P07195	L-lactate dehydrogenase B chain	35	14	118	23
P11279	Lysosome-associated membrane glycoprotein 1	3	2	10	5
P32119	Peroxiredoxin-2	107	18	282	18
P30041	Peroxiredoxin-6	41	13	95	18
Q13492	Phosphatidylinositol-binding clathrin assembly protein	1	1	18	13
P00558	Phosphoglycerate kinase 1	46	20	102	28
F2Z2J9	Phosphoglycerate mutase	1	1	55	15
P08397	Porphobilinogen deaminase	55	18	111	22
Q9UKV8	Protein argonaute-2	6	4	43	22
P00491	Purine nucleoside phosphorylase	67	16	141	21
P30613	Pyruvate kinase isozymes R/L	17	13	97	32
P50395	Rab GDP dissociation inhibitor beta	54	28	132	40
Q96NA2	Rab-interacting lysosomal protein	2	2	27	14
Q99986	Serine/threonine-protein kinase VRK1	41	20		
F5GWT4	Serine/threonine-protein kinase WNK1	1	1	24	20
A6NKH4	Sorting nexin 1			8	7
B4DEK4	Sorting nexin 2	1	1	16	13
Q9NRS6	Sorting nexin-15			3	3
Q9Y5X3	Sorting nexin-5	1	1	4	4
Q9UNH7	Sorting nexin-6	2	1	10	9
Q9Y5X1	Sorting nexin-9			4	3
Q9H2G2	STE20-like serine/threonine-protein kinase	1	1	22	16
P31948	Stress-induced-phosphoprotein 1	28	17	101	39
P17987	T-complex protein 1 subunit alpha	43	22	122	31
P37837	Transaldolase	44	20	74	26
P29401	Transketolase	42	14	117	33
P60174	Triosephosphate isomerase	44	15	104	23
P29144	Tripeptidyl-peptidase 2	1	1	78	45
P54578	Ubiquitin carboxyl-terminal hydrolase 14	18	9	87	27
Q9C0C9	Ubiquitin-conjugating enzyme E2 O	5	3	77	37
Q96RL7	Vacuolar protein sorting-associated protein 13A			11	9
F5GYF5	Vacuolar protein sorting-associated protein 35	1	1	14	11

Sorted populations of reticulocytes and extruded nuclei were fractionated by 1D SDS-PAGE and subjected to Nano LC mass spectrometry. An abridged list containing key cytosolic and endocytic proteins of interest is shown. Total peptide column is the total number of peptides (and therefore an indication of a particular protein’s abundance) detected in the population, whilst the unique peptide column indicates the number of unique peptides detected. To assess differences between nuclei and reticulocyte populations the total peptide number should be used.

The extruded nuclei population was enriched for peptides from nuclear proteins (e.g. histones, lamins, DNA topoisomerase, nuclear pore proteins), ER proteins (e.g. PDI, calnexin, calreticulin), and a number of membrane proteins (e.g. integrins). Generally, low numbers of erythrocyte membrane protein peptides were detected in the nuclei but equal numbers of peptides for several membrane proteins including stomatin, transferrin receptor, Na^+^K^+^ ATPase and basigin were detected in both the nuclei and reticulocyte samples. Interestingly, peptides from actin and actin binding proteins (e.g cortactin, actinin, ARP 2/3 components) were also detected in the nuclei population, suggesting that some actin and associated proteins are lost with the nucleus at this stage, reflecting their additional role in nuclear processes [16]. It is notable that although higher numbers of peptides for band 3, CD44, GPC, Glut1 and Aquaporin1 were detected in the reticulocytes, considerable numbers of peptides for these proteins were also detected in the nuclei sample. Overall this proteomic dataset confirms the enrichment of erythroid membrane proteins to the reticulocyte and also reflects the fact that the extruded nucleus contains ER proteins, a proportion of cytosol and is surrounded by plasma membrane.

### Distribution of Membrane Proteins in Reticulocyte and Nuclei Populations by Western Blotting

**Figure 2:**
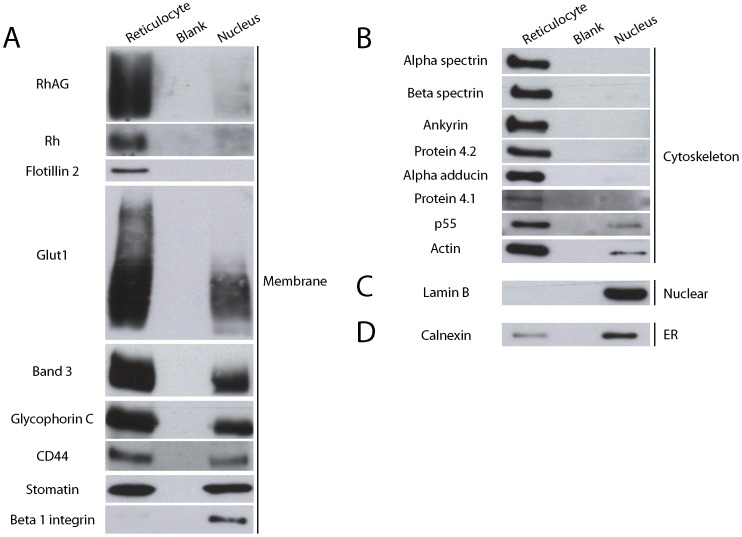
Erythroid protein distribution in sorted populations of reticulocytes and extruded nuclei. Sorted populations of extruded nuclei and reticulocytes were lysed and either 5×10^5^ or 1×10^6^ reticulocytes and nuclei were loaded depending on the protein expression levels or antibody sensitivity. Western blotting was conducted on A) membrane proteins using a mouse monoclonal antibody to Band 3, rabbit polyclonals to RhAG, Rh, Flotillin-2, Glut1, GPC, CD44 and stomatin and a rabbit monoclonal to beta 1 integrin B) cytoskeletal proteins using mouse monoclonal antibodies to alpha spectrin, beta spectrin, ankyrin, protein 4.2 and actin and rabbit polyclonals to alpha adducin, protein 4.1 and p55. C) nuclear protein Lamin B using a goat polyclonal. D) ER protein calnexin using a monoclonal antibody. Blots for RhAG, Rh, band 3, GPC, CD44, alpha spectrin, beta spectrin, ankyrin, protein 4.2 and lamin B are representative of 3–4 repeats from 3–4 independent cultures and sorting experiments. Blots for flotillin-2, Glut1, stomatin, beta 1 integrin, alpha adducin, protein 4.1, p55, actin and calnexin are representative of 2 repeats from 2 independent cultures and sorting experiments. All western blots shown were conducted on material isolated from the same reticulocyte and nuclei sorting experiment.

The partitioning observed for key membrane and cytoskeletal proteins using proteomics was confirmed by western blotting (Figure 2A–B). Importantly, cytoskeletal proteins (alpha and beta spectrin) or cytoskeletal adaptor proteins (ankyrin, 4.1, adducin, protein 4.2) were clearly restricted to the reticulocyte. Interestingly two components of the erythroid cytoskeleton, p55 and actin, were not totally restricted (Figure 2B). Lamin B, a protein of the nuclear lamina, was found only in the nuclei illustrating the purity of the reticulocyte and nuclei populations (Figure 2C). Nuclei contained high levels of the ER protein calnexin consistent with the loss of the majority of the ER with the nucleus (Figure 2D). Some membrane proteins (e.g. Rh and RhAG) were barely detectable in the nuclei population by western blot, highlighting the sensitivity of the mass spectrometry approach and the heightened retention of these proteins in reticulocytes. Importantly, we consistently detected significant amounts of band 3, GPC, CD44 and Glut1 in both reticulocyte and nuclei samples highlighting differential retention of specific membrane proteins to the reticulocyte during the enucleation process. This work also highlights enucleation as a significant stage of stomatin loss, since stomatin partitioned equally between reticulocyte and nuclei populations whereas another lipid microdomain protein, flotillin-2, was restricted to reticulocytes (Figure 2A).

### Imaging Protein Distribution during Early and Late Stages of Enucleation

**Figure 3:**
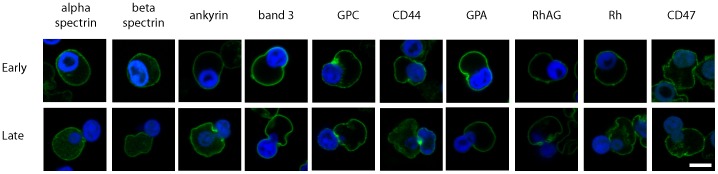
Immunofluorescence of membrane and cytoskeletal protein localisation during human erythroblast enucleation. Human orthochromatic erythroblasts undergoing enucleation after 144 h of differentiation were removed from culture, fixed in 0.5% acrolein and permeabilised using 0.05% Triton X-100. Images shown are slices through cells in early (upper row) and late stages (lower row) of the enucleation process and detected with monoclonal antibodies against alpha spectrin, beta spectrin, ankyrin, band 3, GPC, GPA, RhAG, Rh, CD47 and a rabbit polyclonal antibody against CD44 and a suitable species specific fluorescent secondary as described in materials and methods. N = 5 for each antibody (although generally between 5–20) except for beta spectrin due to problems with high background fluorescence in the nucleus. Scale bar = 5 µm.

**Figure 4:**
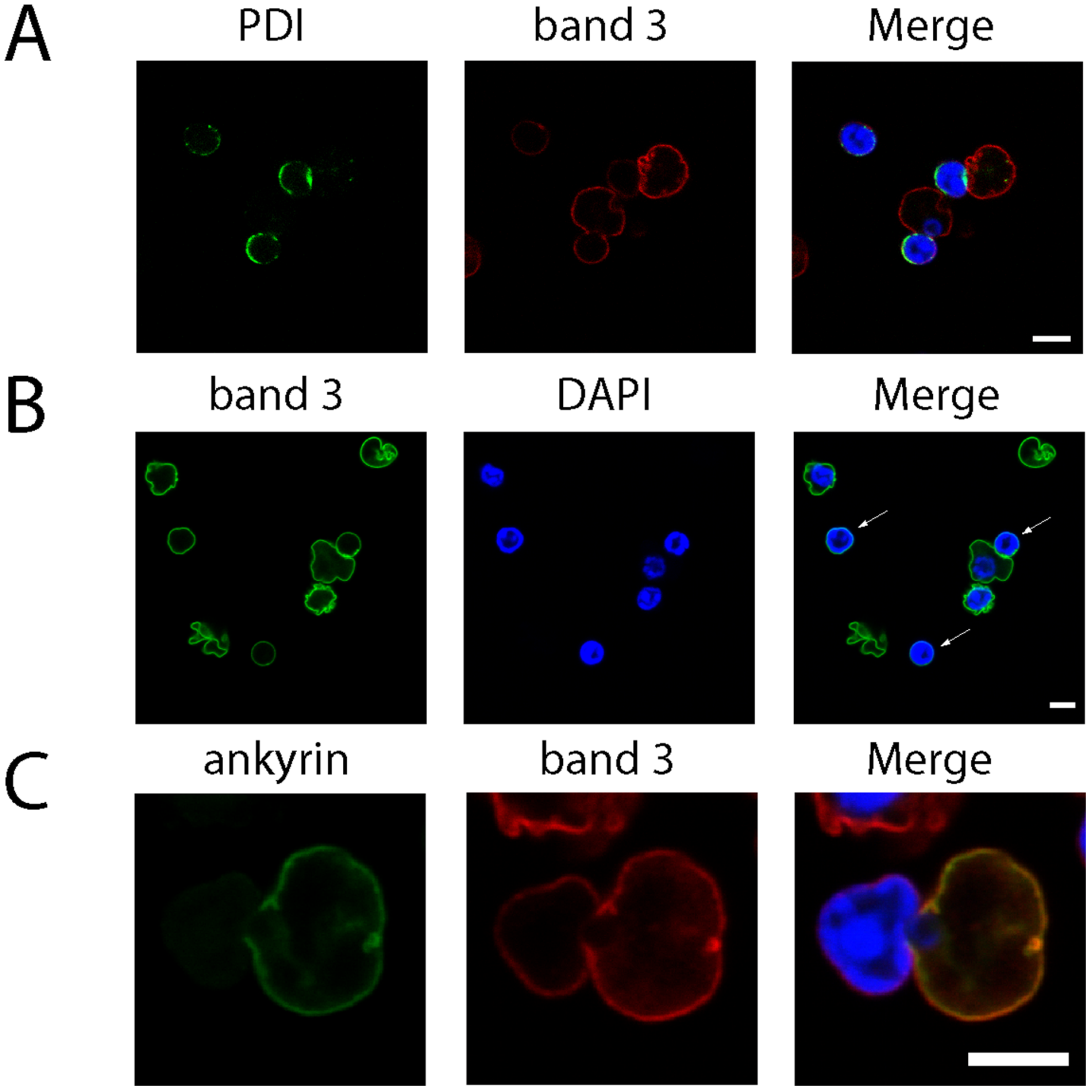
Immunofluorescence microscopy confirms that band 3 and ER are lost during nuclear extrusion. Human orthochromatic erythroblasts undergoing enucleation after 144 h of differentiation were removed from culture, fixed in 0.5% acrolein and permeabilised using 0.05% Triton X-100. A) Confocal section of enucleating cells labelled with PDI and band 3 antibodies. B) Confocal section showing extruded nuclei (marked with arrows) labelled with band 3 (BRIC170). C) Confocal section of an erythroblast in the late stage of enucleation co-labelled with ankyrin and band 3 antibodies. The non-association of band 3 with ankyrin was observed in every cell identified at the late stage of enucleation (n = 10). Scale bar represents 5 µm.

To investigate the localisation and distribution of cytoskeletal and membrane proteins during enucleation, confocal imaging of acrolein fixed erythroblasts was undertaken (Figure 3). No obvious change in membrane protein distribution was observed during the early stages of nuclear extrusion (top row, Figure 3) where the nucleus has polarised and begins to deform the membrane. However, in cells where the nucleus is being deformed as it is squeezed out of the cell (bottom row, Figure 3), complete or partial partitioning of certain erythroid membrane proteins (GPA, GPC, Rh, RhAG and CD47) and cytoskeletal proteins/cytoskeleton associated proteins (alpha spectrin, beta spectrin and ankyrin) to the reticulocyte was observed. We conclude that remodelling of the cytoskeleton and of the majority of membrane components occurs during the late stages of enucleation. Confocal imaging of the ER protein Protein Disulphide Isomerase (PDI) confirmed that ER membrane surrounding the nucleus partitions with the nuclei (Figure 4A). Although the majority of the PDI staining localised as a ring around the nucleus, some remnants of PDI were observed in nascent reticulocytes (results not shown) further supporting the distribution of calnexin shown by Western blot in Figure 2D.

Interestingly, Figure 3 shows by immunofluorescence that band 3 and CD44 were distributed evenly around the plasma membrane surrounding both the reticulocyte and nucleus throughout enucleation. Band 3 was also detected on isolated extruded nuclei (Figure 4B). However, proteins which connect band 3 to the spectrin cytoskeleton (protein 4.2 and ankyrin) together with other membrane proteins located within band 3 multiprotein complexes (Rh, RhAG, CD47, GPA) were largely excluded from the extruding nucleus as illustrated in Figure 3 and by the co-labelling of band 3 and ankyrin (Figure 4C).

## Discussion

We have provided the most detailed study to date of the protein distribution between reticulocytes and the extruded nuclei. This has confirmed that many erythroid membrane and cytoskeletal proteins partition predominantly or exclusively to the reticulocyte during this process. In contrast, nuclear proteins, ER proteins, and a contingent of cytosolic and plasma membrane proteins distribute with the extruded nucleus. This is consistent with observations using electron microscopy where the extruded nucleus is described as being accompanied by a thin rim of cytoplasm, surrounded by plasma membrane [17], [18]. Furthermore, we have demonstrated here that the majority of the ER is lost with the extruded nucleus, building on the observation by imaging that the ER protein calreticulin is lost with the nucleus [11]. ER remnants are still detectable by western blotting (see Figure 2D) and by confocal imaging (results not shown) in the reticulocyte, which we presume are lost upon further reticulocyte maturation.

This work highlights the enucleation step as a significant point of membrane remodelling in human erythropoiesis where excess erythroid membrane proteins are discarded. Unlike in mouse erythroblasts [5], [6], a significant population of human band 3 and to a lesser extent GPC is lost during enucleation. The apparent disparity in distribution, particularly for band 3, between species during enucleation may be due to intrinsic differences in the membrane protein complex composition known to exist between mice and humans [10] or due to mechanistic differences in the process of erythroblast protein sorting. Other membrane proteins were lost during enucleation including CD44, Glut1 and stomatin. For CD44, this further compounds the loss observed in human *in vitro* cultures during terminal differentiation [19] and since CD44 can bind ankyrin, this additional loss may result from continued competition for ankyrin binding sites with the band 3 population.

It is interesting that several of the proteins lost with the nucleus during enucleation are located in membrane protein complexes. Glut1 and band 3 interact *in vitro* [20] and stomatin interacts with the C-terminus of Glut1 [21]. Similarly an association may also exist between the GPC and p55 [22] observed in the nuclei population. The loss of these proteins with the nucleus, taken in conjunction with the restriction of the majority of membrane proteins to the reticulocyte, suggests that these represent proteins/complexes that are most likely synthesized in excess which are not attached to the cytoskeleton (e.g. by incorporation into ankyrin or junctional complexes) leaving them vulnerable to loss during enucleation. In addition, low numbers of peptides were detected in the nuclei relative to the reticulocytes for several cytosolic enzymes (e.g. 6-phosphofructokinase and calpain; see Table 4), therefore a mechanism may also exist for segregation of certain key cytosolic proteins in the reticulocyte, perhaps by incorporation into membrane/cytoskeletal complexes.

In summary, isolated pure populations of human reticulocytes and nuclei have been used to study protein partitioning during human erythroblast enucleation. This work is the first reported proteomic dataset for reticulocytes and extruded nuclei and provides the foundations for investigating reticulocyte maturation, sorting defects in human erythrocyte membrane disorders, and for comparison of protein sorting using erythroblasts produced using other cell sources (e.g. iPS or embryonic stem cells). Our observations here during human enucleation are generally supportive of the hypothesis that the cytoskeleton plays an important part in the segregation of membrane proteins to the reticulocyte during enucleation. Nevertheless in humans the partitioning and retention of specific proteins including the abundantly expressed band 3 to the reticulocyte, occurs in a less definitive manner than observed in mice. Further studies are needed to establish whether the loss of proteins during enucleation in human erythroblasts is an active or passive process and to ascertain whether disruption of the cytoskeleton, mimicking that of hereditary anaemias, leads to additional loss of proteins in humans in the same manner as has been reported in mice.
